# Tumour‐associated macrophages as a novel target of VEGI‐251 in cancer therapy

**DOI:** 10.1111/jcmm.15421

**Published:** 2020-05-26

**Authors:** Xinhuai Dong, Xuan Huang, Zhicheng Yao, Yun Wu, Delin Chen, Chahui Tan, Jiajie Lin, Danrui Zhang, Yiwen Hu, Jueheng Wu, Guohong Wei, Xun Zhu

**Affiliations:** ^1^ Key Laboratory of Tropical Disease Control (Sun Yat‐sen University) Ministry of Education Guangzhou China; ^2^ Department of Center for Translational Medicine Shunde Hospital Southern Medical University Foshan China; ^3^ Department of Obstetrics and Gynecology Fetal Medicine Center The First Affiliated Hospital of Sun Yat‐sen University Guangzhou China; ^4^ Department of General Surgery The Third Affiliated Hospital of Sun Yat‐sen University Guangzhou China; ^5^ Department of Microbiology Zhongshan School of Medicine Sun Yat‐sen University Guangzhou China; ^6^ School of Basic Medical Science Southern Medical University Guangzhou China; ^7^ Department of Clinical Medicine Zhongshan School of Medicine Sun Yat‐sen University Guangzhou China; ^8^ Department of Basic Medicine Zhongshan School of Medicine Sun Yat‐sen University Guangzhou China; ^9^ Changsha Customs District P.R. China Changsha China; ^10^ Department of Endocrinology The First Affiliated Hospital of Sun Yat‐sen University Guangzhou China

**Keywords:** apoptosis, ASK1 signalling, TAMs, VEGI 251

## Abstract

Tumour‐associated macrophages (TAMs), which possess M2‐like characters and are derived from immature monocytes in the circulatory system, represent a predominant population of inflammatory cells in solid tumours. TAM infiltration in tumour microenvironment can be used as an important prognostic marker in many cancer types and is a potential target for cancer prevention or treatment. VEGI‐251 not only is involved in the inhibition of tumour angiogenesis, but also participates in the regulation of host immunity. This work aimed to investigate the involvement of VEGI‐251 in the regulation of specific antitumour immunity. We found that recombinant human VEGI‐251(rhVEGI‐251) efficiently mediated the elimination of TAMs in tumour tissue in mice, and induced apoptosis of purified TAMs in vitro. During this process, caspase‐8 and caspase‐3 were activated, leading to PARP cleavage and apoptosis. Most importantly, we further elucidated the mechanism underlying VEGI‐251‐triggered TAM apoptosis, which suggests that ASK1, an intermediate component of the VEGI‐251, activates the JNK pathway via TRAF2 in a potentially DR3‐dependent manner in the process of TAM apoptosis. Collectively, our findings provide new insights into the basic mechanisms underlying the actions of VEGI‐251 that might lead to future development of antitumour therapeutic strategies using VEGI‐251 to target TAMs.

## 1 INTRODUCTION

Macrophages are phagocytic cells differentiated from monocytes that emigrate from blood vessels to disseminate throughout the body and reside in various tissues, contributing to host defence and tissue repair and remodelling.^1^ Macrophages are conventionally classified into two subtypes with distinct polarization states, namely the M1 and the M2 macrophage phenotypes.^1^ M1 macrophages are pro‐inflammatory, and in contrast, M2 macrophages are anti‐inflammatory.^1^ Accumulating evidence suggests that most malignant tumours contain macrophages with M2‐like phenotype, namely tumour‐associated macrophages (TAMs), as a major component of their microenvironment.^2^, ^3^ Of note, TAMs represent a predominant population of inflammatory cells in the microenvironment and tissue of human solid tumours; in addition, in response to stimulation, the TAMs release a range of growth factors, inflammatory cytokines and proteolytic enzymes that play pivotal roles in tumorigenesis.^2^, ^3^ Furthermore, TAMs contribute to cancer progression and metastasis at different levels, through various mechanisms including promotion of genetic instability, poor tumour antigen presentation, enhanced angiogenesis, generation of cancer stem cells, facilitation of metastasis and other properties.^2^, ^3^ Therefore, TAM infiltration might be an indicator for clinical prognosis and outcome in human cancers and a potential therapeutic target as well.

Increasing evidence indicates that there are numerous potential antitumour therapies involving TAMs.^2^, ^3^ These strategies are grouped into at least five categories by their mechanism of action: (I) eliminating TAM in tumour microenvironment; (II) blocking macrophage recruitment; (III) promoting the M1 phenotype tumoricidal activity of TAMs, which means reprogramming TAMs into pro‐inflammatory M1 macrophages; (IV) suppressing the M2 phenotype tumour promoter activity of TAMs; and (V) using TAMs to deliver anticancer drugs into the tumour environment or targeting TAMs with tumour‐directed monoclonal antibodies that elicit extracellular killing or phagocytosis of cancer cells.^2^, ^3^ For instance, trabectedin, which has been used to treat human ovarian cancer and myxoid liposarcoma in clinic, mechanistically acts by blocking macrophage recruitment through the targeting of monocyte chemoattractant protein‐1 (MCP‐1/CCL2), which correlates with increased macrophage infiltration.^4^, ^5^, ^6^

It has been well documented that vascular endothelial growth inhibitor (VEGI) is a potent endogenous inhibitor of angiogenesis,^7^, ^8^, ^9^ expressed in three isoforms, that is VEGI‐174, VEGI‐192 and VEGI‐251 (also named TNF ligand‐related molecule 1A, or TL1A), which have been found to display various degrees of antitumour or antiangiogenic activity.^7^, ^8^, ^9^ The difference in the isoforms lies in the amino acid sequences of their N‐terminal regions, whereas they share the same 151‐residue C‐terminal segment.^7^ VEGI is a potential therapeutic agent in anti‐angiogenesis‐based cancer treatment because it can induce endothelial cell apoptosis.^10^, ^11^, ^12^, ^13^, ^14^ In our two previous studies, we developed an improved method to produce recombinant VEGI (rhVEGI‐192) and provided the first demonstration that VEGI‐192 can form a polymeric structure, which may account for its antiangiogenic activity.^8^ In addition, we developed a promising enhanced antiangiogenic and antitumour strategy by using the application of the RGD (a ligand of αvβ3 integrin)‐VEGI‐192 fusion protein, leading to a combination therapy against multiple tumour‐targets.^9^ It is well established that VEGI‐251 not only inhibits tumour angiogenesis but also regulates host immunity. Studies have revealed VEGI‐251 could play a role as a co‐stimulator of T cells in mediating inflammation.^15^, ^16^ The main receptor for VEGI‐251, among the three VEGI isoforms, has been clearly identified as death receptor 3 (DR3) (designated TNFRSF25).^16^, ^17^ Currently, available data also provide evidence that DR3 is expressed on a variety of cell types, including macrophages, endothelial cells, natural killer (NK) cells and lymphocytes.^18^, ^19^, ^20^ The complex of VEGI‐251 and DR3 could recruit adaptor proteins, including TNF receptor–associated factor 2 (TRAF2) and tumour necrosis factor receptor type 1–associated DEATH domain protein (TRADD), to activate c‐Jun N‐terminal kinase (JNK) and trigger the caspase‐3‐mediated apoptosis pathway,^17^ but the mechanism by which the VEGI‐251‐DR3 complex activates JNK signalling remains to be identified.

This work aimed to investigate the involvement of VEGI‐251 in the regulation of specific antitumour immunity. We explored the effects of VEGI‐251 on TAMs and the underlying mechanisms using an animal tumour model. This study showed that recombinant human VEGI‐251 (rhVEGI‐251) induced apoptosis of TAMs. During this process, caspase‐8 and caspase‐3 are activated, leading to PARP cleavage and apoptosis. We found that via binding to the DR3 receptor on the membrane of TAMs, VEGI‐251 facilitates the formation of the DR3‐TRAF2 protein complex. Then, this complex can directly activate the kinase activity of apoptosis signal‐regulating kinase 1 (ASK1) and, subsequently, c‐Jun transcriptional activity and downstream Puma expression, ultimately inducing the apoptosis of TAMs. Our findings collectively revealed that beyond the antiangiogenic and direct tumour cell‐killing activities of VEGI‐251, TAMs might be a novel target of VEGI‐251 for cancer therapy.

## 2 MATERIALS AND METHODS

### 2.1 Preparation of rhVEGI‐251 protein and mass spectrometry analysis

Coding sequence for full‐length VEGI‐251 was amplified by reverse transcription‐polymerase chain reaction (RT‐PCR) by using HUVEC (human umbilical vein endothelial cell) mRNA as the template. The primer sequences as shown in supplemental Table S1 included the restriction sites Kpn I and Bam HI for PCR‐mediated cloning into the eukaryotic expression vector pEF‐BOS (Invitrogen, Carlsbad, CA). A 6× His‐Tag sequence was introduced into the protein sequence at the carboxy‐terminus. To obtain the VEGI‐251‐His protein, the expression and purification of rhVEGI‐251 protein were performed in accordance with an established method as previously described^8^, ^9^ via Ni‐NTA affinity chromatography (The QIAexpressionist, Qiagen, Chatsworth, CA, USA). The vehicle control was the product extracted by expression of the empty pEF‐BOS vector without VEGI‐251 using the same expression and purification protocol as described above. After the purified rhVEGI‐251, protein was separated by SDS‐PAGE (sodium dodecyl sulphate polyacrylamide gel electrophoresis) and assessed with silver staining using a ProteoSilver Plus Silver Staining Kit (Sigma‐Aldrich, St. Louis, MO, USA), and then, the clear single target band was excised from the gel and subjected to mass spectrometry analysis.

### 2.2 Cell culture and cell viability analysis

Unless otherwise stated, all laboratory chemicals, reagents and disposable labware were purchased from Oxoid (Basingstoke, Hampshire, UK), Sigma‐Aldrich or Sangon Biotech (Shanghai, China). HUVECs were maintained in serum‐free Human Endothelial‐SFM (GIBCO, Carlsbad, CA, USA), supplemented with endothelial cell growth supplement at a final concentration of 15 g/mL (Upstate, Billerica, MA, USA). Hep1‐6 cells were cultured in Dulbecco's modified Eagle's medium (DMEM) (Invitrogen, Carlsbad, CA, USA), and TAMs and THP1 were cultured in RPMI‐1640 (Gibco, Carlsbad, CA, USA) supplemented with 10% foetal bovine serum (FBS) (Gibco), 100 units/ml penicillin and 100 μg/mL streptomycin (Invitrogen), and 2 mmol/L L‐glutamine (Invitrogen). THP‐1 monocytic cells were differentiated with phorbol‐12‐myristate‐13‐acetate (PMA, 100ng/mL, Sigma‐Aldrich, St. Louis, MO) for 24 hours, followed by a resting period of 6 days, and then, these resting differentiated macrophages were treated with 30 ng/mL of IL‐4 (R&D Systems, Minneapolis, MN, USA) for 24 hours to obtain M2‐like macrophage phenotypes, as previously described.^21^ The small interfering RNA (siRNA) targeting mouse ASK1, and mouse DR3, and scrambled control siRNA were purchased from RiboBio Co., Ltd. (Guangzhou, China), and targeting sequences are provided in supplemental Table S1. Transfection of the plasmids and siRNAs at the indicated concentrations was performed using Lipofectamine 2000 reagent (Invitrogen) according to the manufacturer's instructions. All cells were cultured at 37°C in a humidified atmosphere containing 5% CO_2_.

The effect of rhVEGI‐251 protein on the growth of HUVECs was assessed with a 3‐(4,5‐dimethylthiazol‐2‐yl)‐2,5‐diphenyl tetrazolium bromide (MTT) assay by exposing cells to various amounts of rhVEGI‐251 preparation for 48 hours as previously described.^8^, ^9^ The absorbance was measured at 490 nm using a microplate reader (Bio‐Tek Synergy 2, Winooski, VT, USA). Cell growth inhibition was determined using the following formula according to a previously published method: growth inhibition (%) = (1−OD of treated cells/OD of control cells) × 100%, where OD is the optical density.^9^, ^22^ The half maximal inhibitory concentration (IC_50_) was calculated with Bliss's software,^9^, ^22^ and the data were analysed in SPSS software. One unit (U) of rhVEGI‐251 protein activity is defined as the IC_50_ value; that is, the concentration of rhVEGI‐251 protein required to decrease the growth rate of HUVECs by 50%, as previously described.^12^ All experiments were performed for three times, and the mean values were calculated from the triplicate experiments.

### 2.3 Animal tumour model and antitumour effect of rhVEGI‐251 in vivo

All animal experimental procedures were approved by the Institutional Animal Care and Use Committee of Sun Yat‐sen University. C57BL/6J mice (16‐20 g, 5‐6 weeks of age) were purchased from the SLAC Laboratory Animal Co., Ltd. (Shanghai, China) and were housed in a barrier facility on a 12‐h/12‐h light/dark cycle. On day zero, Hep1‐6 cells (3 × 10^6^ cells in 0.1 mL/mouse) were inoculated subcutaneously in the necks of the mice as previously described.^9^, ^22^ Before treatment, eighteen mice were ear‐tagged and randomly divided into one control group (n = 6) and two treatment groups (n = 6 for each group) in a blinded manner. From day six, the formed transplanted tumours were measured every four days. On day six, animals in the two treatment groups received an intraperitoneal (ip) dose of either 5 mg/kg or 10 mg/kg bodyweight rhVEGI‐251 in a 100 μL volume every four days, whereas each mouse in the vehicle control group received a ip injection of 100 μL of vehicle control preparation. The perpendicular diameters of the tumours were measured every four days with a digital calliper, and tumour volumes (mm^3^) were calculated as width × width ×length × π/6. At the endpoint of the animal experiment, all the mice were killed, and the tumours were harvested and weighed. Data are presented as the means ± standard deviations (SD) in each group.

### 2.4 TAM isolation and identification by flow cytometry analysis

Fresh tumour tissues from the above‐mentioned homograft tumour model were used for the isolation of tumour‐associated macrophages as previously described.^23^ For the isolation of tumour‐infiltrating mononuclear cells, freshly obtained tumour tissues were cut into small pieces and then suspended in RPMI 1640 medium containing 10 mg/mL DNase I (Roche, Basel, Switzerland) and 1 mg/mL collagenase IV (Sigma‐Aldrich), followed by gentle mechanic dissociation using a Tumor Dissociation Kit (Miltenyi Biotec, Bergisch Gladbach, Germany) according to the manufacturer's protocol. Suspensions of dissociated cells were further incubated for 1 hour at 37°C with continuous rotation and were then filtered through 70‐μm cell strainers to obtain single‐cell suspensions. For the isolation of TAMs, single‐cell suspensions were washed in PBS supplemented with 2% FCS and adjusted to a concentration of 1 × 10^7^ cells/mL. Then, the cells were incubated with CD11b MicroBeads™ followed by the instructions provided by the manufacturer and isolated by fluorescence‐activated cell sorting (FACS) analysis in a BD FACSAria III flow cytometer (BD Biosciences, San Jose, CA, USA).

Flow cytometry analysis was used to analyse the surface markers of tumour‐infiltrating mononuclear cells or TAMs. In brief, single‐cell suspensions or purified TAMs were washed in PBS supplemented with 2% FCS and adjusted to a concentration of 1 × 10^7^ cells/mL. Cells were incubated with a fluorescein isothiocyanate (FITC)–conjugated antibody against mouse F4/80 (eBioscience, CA, USA) or a phycoerythrin (PE)‐Cy5‐conjugated antibody against mouse CD11b (eBioscience). The FITC‐conjugated antibody against mouse F4/80 was used at 5 µg/mL, and the PE‐Cy5‐conjugated antibody against mouse CD11b was used at 1.25 μg/mL. Cells were incubated with the indicated antibodies at 4°C for 30 minutes and rinsed with PBS for three times. Then, cell samples were analysed in an EPICS XL‐MCL flow cytometer (Beckman Coulter, Brea, CA, USA), and the data were analysed using FlowJo 7.6 software (TreeStar Inc, Ashland, OR, USA).

### 2.5 TUNEL (Terminal deoxynucleotidyl transferase–mediated dUTP nick end labelling) assay

Apoptotic DNA fragmentation in TAMs induced by rhVEGI‐251 was examined using a DeadEnd™ Fluorometric TUNEL System kit (Promega, Madison, WI, USA) according to the manufacturer's protocol.^22^ In brief, cells (at a density of 1 × 10^5^ cells/well) were seeded on coverslips in 24‐well flat‐bottom plates and treated with 2, 4 or 8 U rhVEGI‐251 for 24 hours. Following treated with rhVEGI‐251, cells were fixed with 4% paraformaldehyde in PBS at 4°C for 30 minutes. Fixed cells were then washed with PBS for three times, permeabilized in 0.1% Triton X‐100 on ice for 2 minutes and labelled with fluorescein‐12‐dUTP for 1 hour by using terminal deoxynucleotidyl transferase at 37°C in a humidified chamber. After three further rinses with PBS, cell nuclei were double‐stained with propidium iodide (PI) at concentration of 1 μg/mL at room temperature for 15 minutes. The localized green (fluorescein‐12‐dUTP) or red (PI) fluorescence of apoptotic cells was detected by fluorescence microscopy (Zeiss Axiovert 100M, Carl Zeiss, Germany). Ten randomly chosen microscopic fields were imaged. Experiments were performed in triplicate.

### 2.6 Caspase activity assay

The activity of caspase‐8 or caspase‐3 in TAMs was examined using a caspase colorimetric assay kit (Keygen Biotech, China) in accordance with the manufacturer's protocol.^22^ In brief, following treatment with rhVEGI‐251 at different concentrations for 24 hours, TAMs were harvested and washed with PBS three times and were then resuspended in chilled 1× lysis buffer.^22^ Subsequently, the cell lysates were centrifuged at 10 000 *g* for 1 minute and incubated on ice for 60 minutes. The supernatants were collected in fresh tubes, and the total protein concentrations were measured with a Bradford protein assay kit (Keygen Biotech, China) according to the manufacturer's instructions.^22^ A total of 150 μg of each sample in a 96‐well plate was diluted with lysis buffer (50 μL) and added to 2× reaction buffer (50 μL) containing 10 mmol/L dithiothreitol (DTT).^22^ Then, 5 μL of a colorigenic substrate was added to each well, and the plate was incubated at 37°C in the dark for 4 hours.^22^ The absorbance at 405 nm was measured using a microplate reader (Bio‐Tek Synergy 2, Winooski, VT, USA). The fold increase in caspase activity was determined by comparing the results with those obtained for the non‐treatment control.

### 2.7 Western blot analysis

After treatment with rhVEGI‐251 at different concentrations for 48 hours, cells were collected and lysed in 1× sample buffer (6% SDS, 50 mmol/L Tris‐HCl (pH 7.4), 10% glycerol, 5% mercaptoethanol, 1 mmol/L PMSF and 0.1% bromophenol blue) and then were lysed by sonication.^9^, ^22^ The concentration of the protein samples was determined by a Bicinchoninic Acid Protein Assay Kit (Thermo Fisher Scientific, Rockford, IL, USA) followed by the manufacturer's instructions, with bovine serum albumin (BSA) as the standard.^9^, ^22^ 40 μg of the total cell lysates was subjected to SDS‐PAGE and transferred to polyvinylidene difluoride (PVDF) membranes. After blocking for 1 hour with Tris‐buffered saline buffer containing 5% non‐fat milk at room temperature, membranes were incubated at 4°C overnight with the following specific primary antibodies: anti‐caspase‐8 (Cell Signaling, Beverly, MA, USA), anti‐caspase‐3 (Cell Signaling), anti‐phospho‐SAPK/JNK (Thr183‐Tyr185) (81E11) (Cell Signaling), anti‐phospho‐c‐Jun (Ser63) (Cell Signaling), anti‐phospho‐c‐Jun (Ser73) (Cell Signaling), anti‐SAPK/JNK, anti‐c‐Jun (60A8), anti‐Puma (Cell Signaling) and anti‐actin (Cell Signaling) antibodies. Further incubation for 1 hour with the horseradish peroxidase (HRP)–conjugated secondary antibody that is appropriate for the primary antibody used was performed at room temperature.^9^, ^22^ The bands and signals were detected with Kodak film using an enhanced chemiluminescence kit (Thermo Fisher Scientific). Actin was used as the loading control for quantitative normalization.

### 2.8 Coimmunoprecipitation assay

Tumour‐associated macrophages were transfected with the indicated plasmids via a standard calcium phosphate coprecipitation method for the indicated time, as previously described.^24^ 24 hours later, cells were lysed with protein lysis buffer (150 mmol/L NaCl, 25 mmol/L HEPES, 2% glycerol, 1 mmol/L EDTA, 1% NP‐40), and protease and phosphatase inhibitor cocktail (Roche). Lysates were incubated at 4°C with IgG antibody or the indicated antibodies overnight. Then, the precipitates were washed five times with wash buffer (150 mmol/L NaCl, 20 mmol/L HEPES, 2% glycerol, 1 mmol/L EDTA, 1 mmol/L EGTA and 0.1% NP‐40), resuspended in sample buffer and subjected to Western blot analysis.

### 2.9 Statistical analysis

All statistical analyses were performed using the SPSS 20.0 statistical software package. The results accorded with normal distribution were expressed as the means ± standard deviations (SD) derived from three independent experiments. Comparisons between two groups were evaluated by a 2‐tailed Student's *t* test. For pairwise multiple comparisons, statistical analyses were performed on data from triplicate experiments using one‐way ANOVA followed by Dunnett's multiple comparison test. *P* values of <0.05 were considered statistically significant.

## 3 RESULTS

### 3.1 Inhibitory activity on endothelial growth and antitumour effect of rhVEGI‐251 in vivo

**FIGURE 1:**
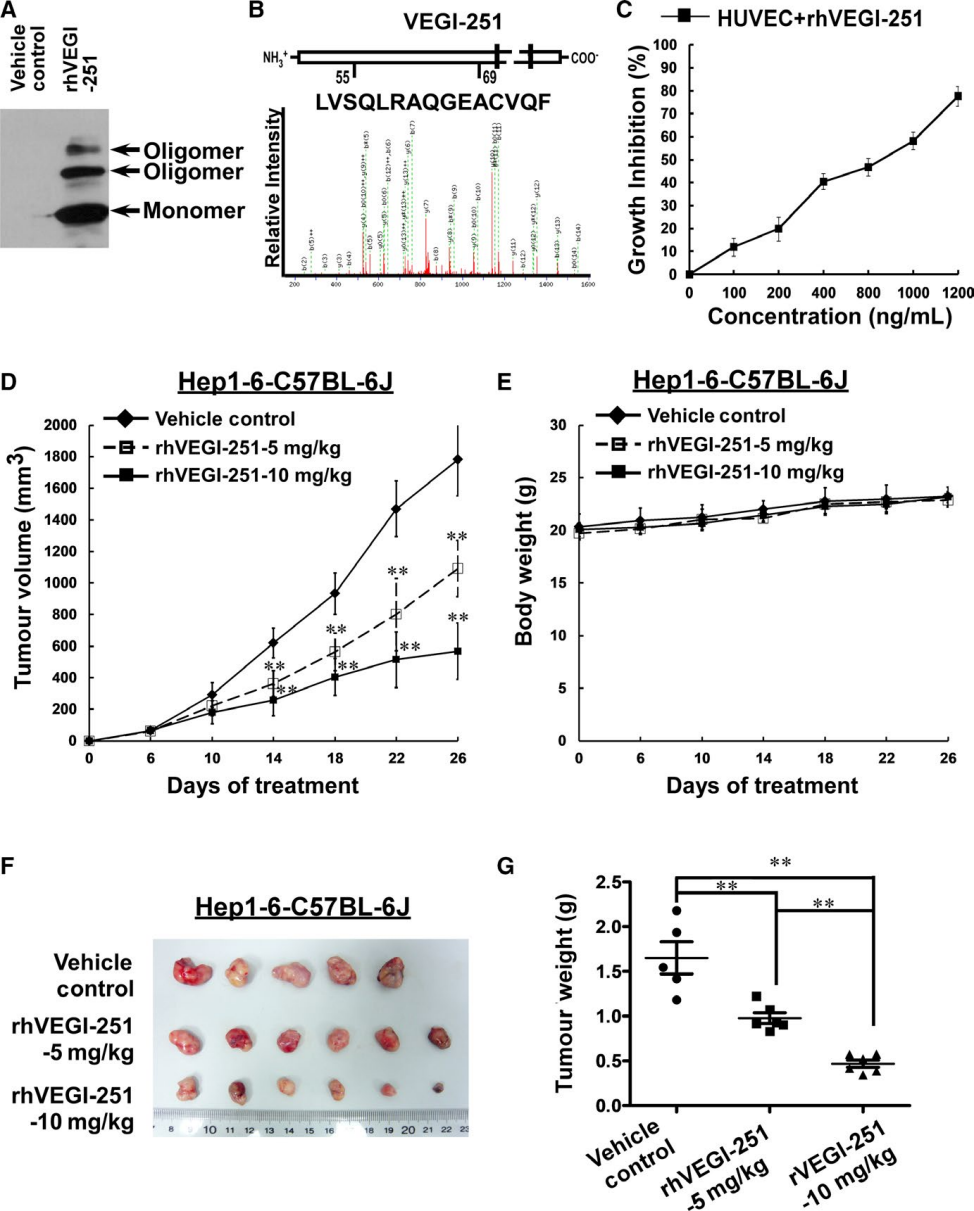
Inhibitory effect of rhVEGI‐251 on endothelial proliferation and tumour growth in vivo. A, Western blot analysis of purified rhVEGI‐251 by non‐reducing SDS‐PAGE. B, Mass spectrometric identification of the purified rhVEGI‐251 products. C, Dose‐response curve of the effect of rhVEGI‐251 on the proliferation of HUVECs after treatment for 48 h. Cell viability was determined by an MTT assay. (D,E,F,G) In vivo antitumour effect of rhVEGI‐251 on Hep1‐6 murine tumours in C57BL‐6J mice. Viable Hep1‐6 cells (3 × 10^6^ cells/mouse) were inoculated subcutaneously into the necks of mice (six mice per group). On day six, mice received an intraperitoneal injection of rhVEGI‐251 (5 mg/kg or 10 mg/kg) or vehicle control preparation every four days. D, Time‐response curve of the effect of rhVEGI‐251 on the growth of tumours formed by Hep1‐6 cells. E, Time‐response curve of the effect of rhVEGI‐251 on the bodyweight of the homograft mice. F, Photographs of the tumour tissues from each group, as indicated (vehicle control: n = 5 mice; 5 mg/kg rhVEGI‐251: n = 6 mice; 10 mg/kg rhVEGI‐251: n = 6 mice). G, Inhibition rate calculated based on the tumour tissue weight for each group. The data accorded with normal distribution are expressed as the means ± SD in each group. One‐way ANOVA followed by Dunnett's multiple comparison test was performed, and significant differences are shown with asterisks (** indicates *P* < 0.01)

rhVEGI‐251 protein was produced by the previously established 293FT eukaryotic expression system and was verified by Western blotting and mass spectrometry; the production method achieved a high degree of purity for the target protein (>95%) (Figure 1A,B). The effect of rhVEGI‐251 protein on the growth of HUVECs was determined by using an MTT assay. Following treatment with varying concentrations of rhVEGI‐251 for 48 hours (Figure 1C), cultured HUVECs exhibited marked dose‐dependent growth inhibition compared with vehicle control cells. The IC_50_ value of rhVEGI‐251 against HUVECs was approximately 750 ng/mL, which was defined as one unit (U).

Then, we evaluated the in vivo anticancer activity of rhVEGI‐251 protein in a murine liver cancer model using Hep1‐6 cells. On day 6 after inoculation, all the tumour volumes in mice were assessed, the results showed all animals had developed tumours (100%). After six doses, marked inhibition of tumour growth was observed (Figure 1D,F,G). In addition, compared to the untreated animals, the treated animals exhibited no signs of behavioural changes, discomfort or significant loss of bodyweight (Figure 1E) on day 26 after rhVEGI‐251 treatment. After injection with different doses of rhVEGI‐251, the volumes of the homograft liver tumours were significantly decreased compared with those in vehicle control‐treated mice (n = 5, one of the original six mice died on the 25th day), by 38.63% in mice treated with 5 mg/kg rhVEGI‐251 (n = 6) and 68.15% in mice treated with 10 mg/kg rhVEGI‐251 (n = 6) (Figure 1D,F). Moreover, on day 26, the weights of the transplanted tumours were significantly decreased by 40.83% and 71.63% in the 5 mg/kg rhVEGI‐251 and 10 mg/kg rhVEGI‐251 groups, respectively (Figure 1G). These results strongly suggested that systemically delivered rhVEGI‐251 could inhibit the growth of established liver tumours in mice in a dose‐dependent manner.

### 3.2 rhVEGI‐251 mediates the elimination of TAMs in tumour tissue

**FIGURE 2:**
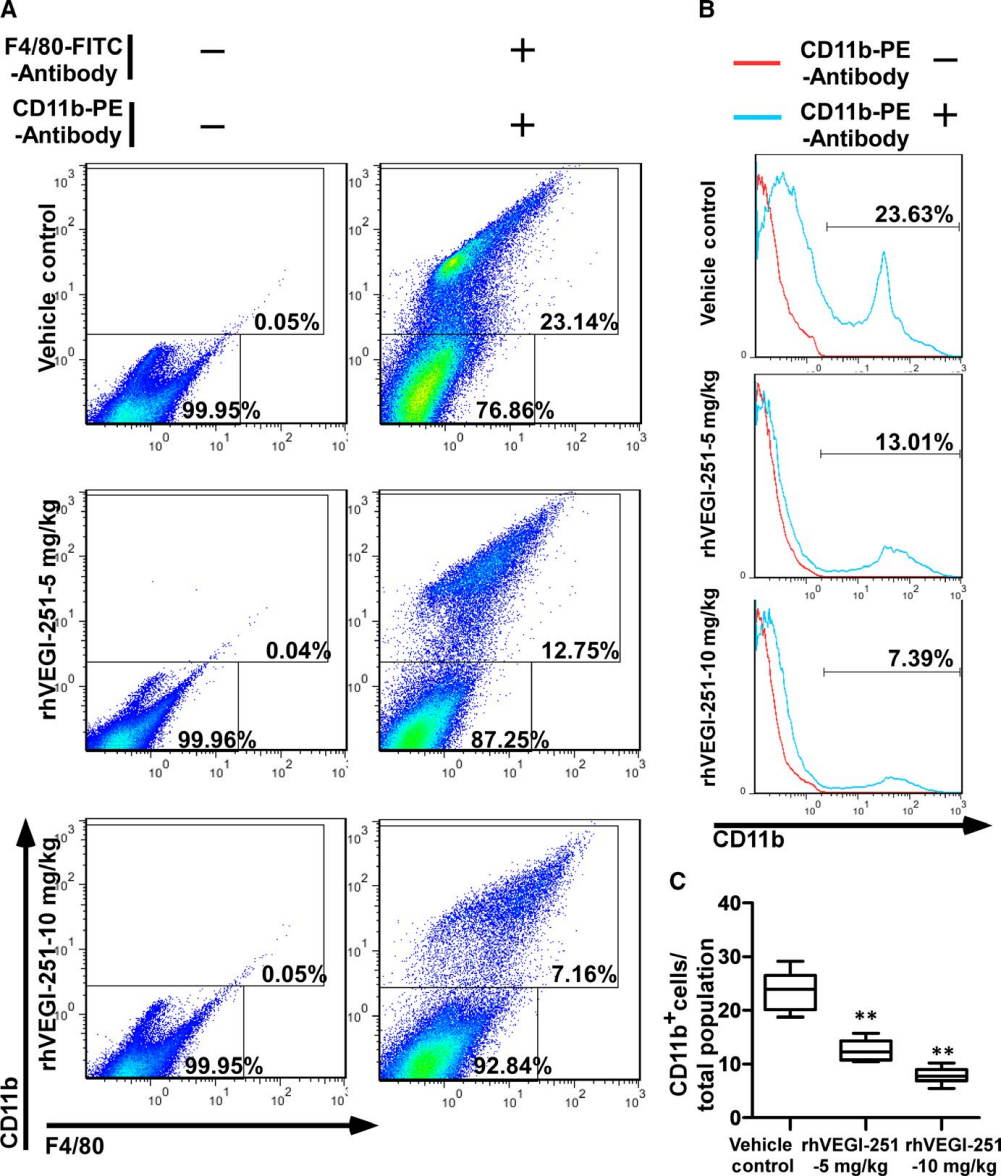
rhVEGI‐251 mediates the reduction of tumour‐infiltrating mononuclear cells in tumour tissue. (A,B) Tumour‐infiltrating mononuclear cells were isolated from fresh tumour and analysed by flow cytometry analysis followed by co‐staining with an anti‐CD11b antibody and an anti‐F4/80 antibody (A) or staining with the anti‐CD11b antibody alone (B). Expression of the indicated cell surface markers (blue) in control (red) CD11b^+^ cells isolated from vehicle control‐treated mice (n = 5), mice treated with 5 mg/kg rhVEGI‐251 protein (n = 6) or 10 mg/kg rhVEGI‐251 protein (n = 6). The images are representative of the results from three independent experiments. C, Statistical analysis of the percentages of CD11b^+^ tumour‐infiltrating mononuclear cells among single cells derived from the tumour tissue of mice. The data accorded with normal distribution are representative of three independent experiments. One‐way ANOVA followed Dunnett's multiple comparison test was performed, and significant differences are shown with asterisks (** indicates *P* < 0.01)

TAMs have been reported to show surface expression of markers such as CD11b and F4/80 in mice.^2^, ^3^, ^25^ Considering accumulating evidence indicating that VEGI‐251 not only is involved in inhibiting tumour angiogenesis but also participates in the regulation of host immunity, and considering that TAM polarization promotes proliferation, migration, invasion and angiogenesis in different types of tumours, we intended to investigate the influence of rhVEGI‐251 on M2‐like TAMs isolated from a tumour in vivo. Tumour‐infiltrating mononuclear cells isolated from freshly obtained tumour tissue from the homograft mouse model formed by Hep1‐6 cells and treated with or without rhVEGI‐251 were analysed by flow cytometry after staining with an antibody against CD11b. After treatment of mice with the vehicle control preparation, 5 mg/kg rhVEGI‐251 protein and 10 mg/kg rhVEGI‐251 protein, 23.56 ± 3.63%, 12.63 ± 1.85% and 7.87 ± 1.45%, respectively, of the isolated single cells in suspension, were identified as CD11b^+^ cells (Figure 2A,B,C). These results showed that the population of CD11b^+^ tumour‐infiltrating mononuclear cells was decreased more in the group of rhVEGI‐251‐treated mice than in the vehicle control‐treated group.

**FIGURE 3:**
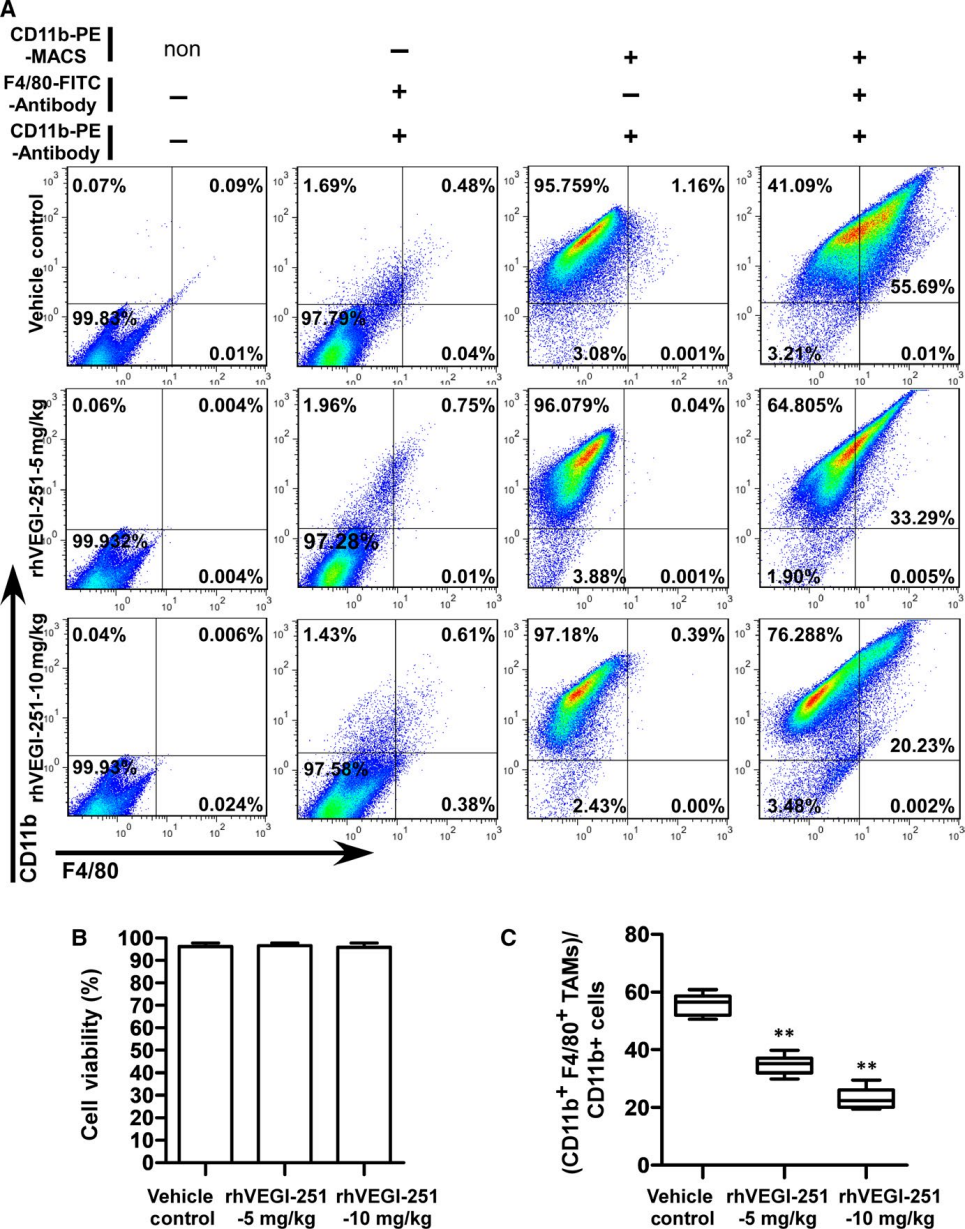
rhVEGI‐251 mediates the elimination of TAMs in tumour tissue. A, Representative results of flow cytometric analysis of CD11b^+^ F4/80^+^ TAMs from CD11b^+^ tumour‐infiltrating mononuclear cells. The data in the first panel were used as a blank control group, and a symbol ‘non’ on the right of CD11b‐PE‐MACS means there were no CD11b MicroBeads added in this group. The data in the second panel represent the negative control group, and a symbol ‘‐’ on the right of CD11b‐PE‐MACS means these cells were collected as flow‐through (wash fractions) after incubated with CD11b MicroBeads, which are CD11b microbeads negative selected cells. The purity of CD11b^+^ cells among all selected cells is shown in the third panel. The proportions of CD11b^+^ F4/80^+^ cells are shown in the fourth panel. The images are representative of the results from three independent experiments. B, The viability of purified TAMs was assessed by a trypan blue exclusion assay. C, Statistical analysis of the percentage of CD11b^+^ F4/80^+^ TAMs among CD11b^+^ tumour‐infiltrating mononuclear cells. One‐way ANOVA followed by Dunnett's multiple comparison test was performed, and significant differences are shown with asterisks (** indicates *P* < 0.01)

The purity of the extracted TAMs in each respective group of mice as listed above was 97.16 ± 1.83%, 96.79 ± 1.03% and 97.24 ± 0.96%, as determined by FACS analysis using an anti‐human CD11b^+^ antibody (Figure 3A). The viability of the purified TAMs derived from each group was assessed by trypan blue exclusion and was repeatedly measured at greater than 95% (Figure 3B). Upon treatment of mice with the vehicle control preparation, 5 mg/kg rhVEGI‐251 protein and 10 mg/kg rhVEGI‐251 protein, 55.63 ± 3.74%, 34.76 ± 3.19%, and 23.23 ± 3.32% of purified TAMs, respectively, were identified as CD11b^+^ F4/80^+^ cells (Figure 3A,C). Meanwhile, an MTT assay was used to verify the function of rhVEGI‐251 on the mouse cancer cell line Hep1‐6. As shown in the Figure S1, we found that rhVEGI‐251 showed no cytotoxicity on Hep1‐6 cells, suggesting rhVEGI‐251 targeted TAMs but not Hep1‐6 cells during systemic administration. These results showed that when treated with rhVEGI‐251, the proportion of TAMs in tumour tissues decreased markedly in a dose‐dependent manner. Taken together, the observed tumour growth inhibition in rhVEGI‐251‐treated mice was accompanied by a decreased proportion of TAMs.

### 3.3 rhVEGI‐251 triggers the apoptosis of TAMs through the activation of extrinsic apoptotic pathways

**FIGURE 4:**
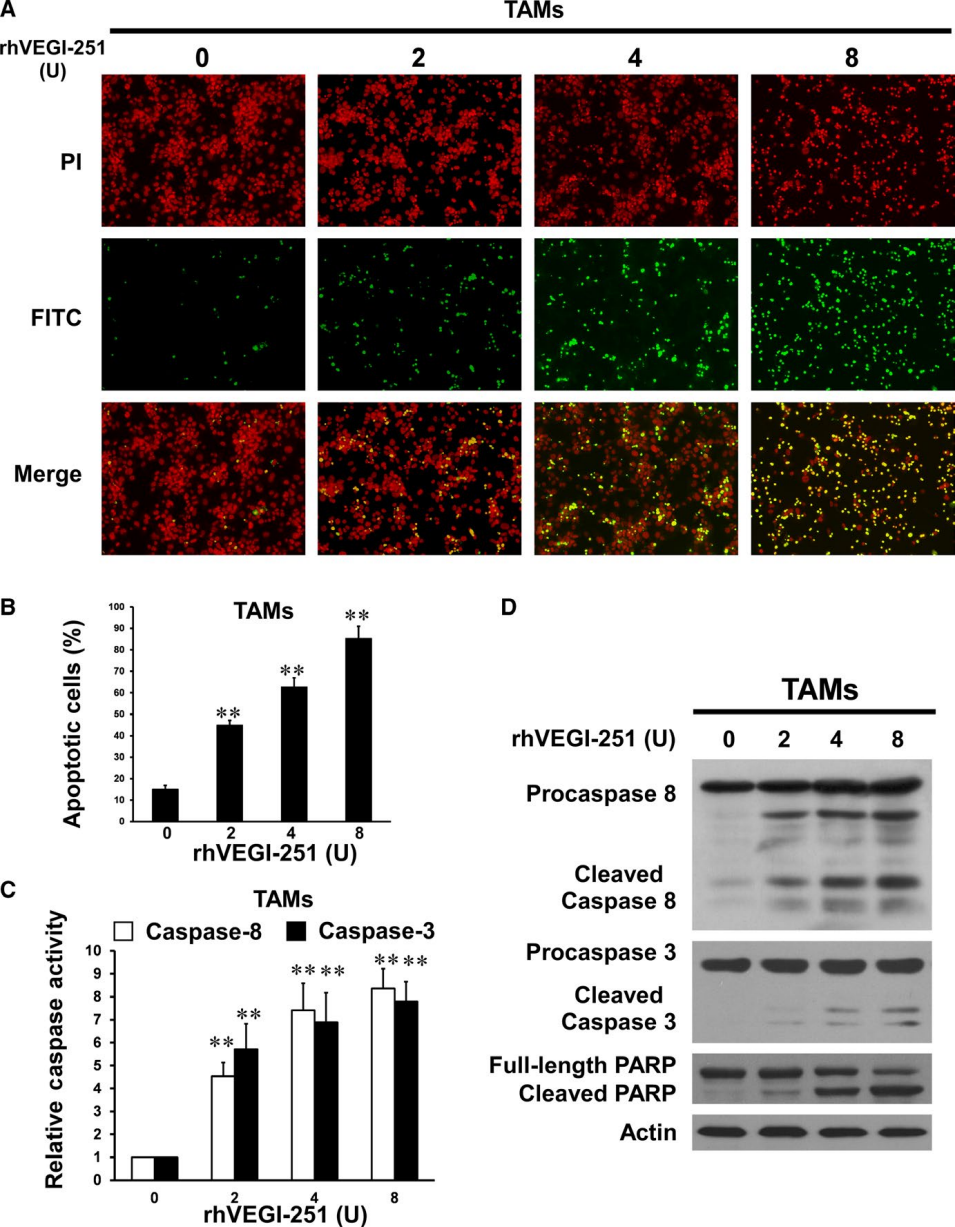
rhVEGI‐251 triggers marked apoptosis of TAMs. A, TAMs were treated with different concentrations of rhVEGI‐251 as noted for 24 h and were then labelled with fluorescein‐12‐dUTP (green) and counterstained with PI (red). B, Apoptotic index, as determined by counting the number and calculating the percentages of TUNEL‐positive cells in 10 fields. Data accorded with normal distribution for the quantitative assessment of apoptosis are expressed as the means ± SD C, Enzymatic activities of caspases in rhVEGI‐251‐treated TAMs, as assessed with a colorimetric assay. The fold increases in the activities of caspase‐8 and caspase‐3 were determined by comparison with those in vehicle‐treated control cells. D, Western blot analysis of caspases and PARP in TAMs after treatment with different concentrations of rhVEGI‐251 for 48 h using antibodies against caspase‐8, caspase‐3 and PARP. Actin was used as the internal control. FITC, fluorescein isothiocyanate; PI, propidium iodide; TUNEL, terminal deoxynucleotidyl transferase–mediated dUTP nick end labelling. The results accorded with normal distribution are presented as the means ± SD One‐way ANOVA followed by Dunnett's multiple comparison test was performed. **P < 0.01 indicates a significant difference compared with the control

To investigate whether the loss of TAMs induced by rhVEGI‐251 was associated with apoptosis, TUNEL assays were employed to assess DNA fragmentation as a late apoptotic event in TAMs. As shown in Figure 4A, more TUNEL‐positive cells were visualized in TAMs treated for 24 hours with rhVEGI‐251 than in vehicle control‐treated cells. In addition, a dose‐dependent pro‐apoptotic response of TAMs to treatment with rhVEGI‐251 was observed. Upon treatment of TAMs with 0, 2, 4 and 8 U of rhVEGI‐251 for 24 hours, 15.04 ± 1.78%, 44.97 ± 2.18%, 62.74 ± 4.27% and 85.45 ± 5.55% of the TAMs, respectively, exhibited chromosomal DNA fragmentation (Figure 4B). Furthermore, the TUNEL assay was applied to show the effect of rhVEGI‐251 on human M2‐like macrophages, which is derived from THP‐1 monocytic cells by treated with PMA and IL‐4. As shown in the supplemental Figure S2, our results suggested that rhVEGI‐251 could also trigger the apoptosis of M2‐like macrophages (THP‐1) in a dose‐dependent manner. Collectively, these results demonstrated that rhVEGI‐251 could induce the apoptosis of TAMs, suggesting that VEGI‐251 might be a pro‐apoptotic cytokine of TAMs in vitro.

To further determine the apoptotic pathway involved in rhVEGI‐251‐triggered apoptosis of TAMs, caspase‐8 and caspase‐3 were examined using colorimetric enzymatic activity assays and Western blot analysis. As shown in Figure 4C, the enzymatic activity of caspase‐8 and caspase‐3 showed a dose‐dependent increase with rhVEGI‐251 protein treatment. Consistent with the results of the colorimetric enzymatic activity assays, rhVEGI‐251 protein treatment dose dependently resulted in activating cleavage of caspase‐8 and caspase‐3, manifested as a significantly increase in the cleaved protein band densities (Figure 4D). In addition, treatment of TAMs with rhVEGI‐251 triggered a dramatic increase in proteolytic cleavage of PARP, a signature event during apoptosis (Figure 4D). Overall, these data demonstrated that TAM apoptosis triggered by rhVEGI‐251 might involve extrinsic pathways.

### 3.4 rhVEGI‐251 activated ASK1/JNK signalling through the interaction of ASK1 with TRAF2

**FIGURE 5:**
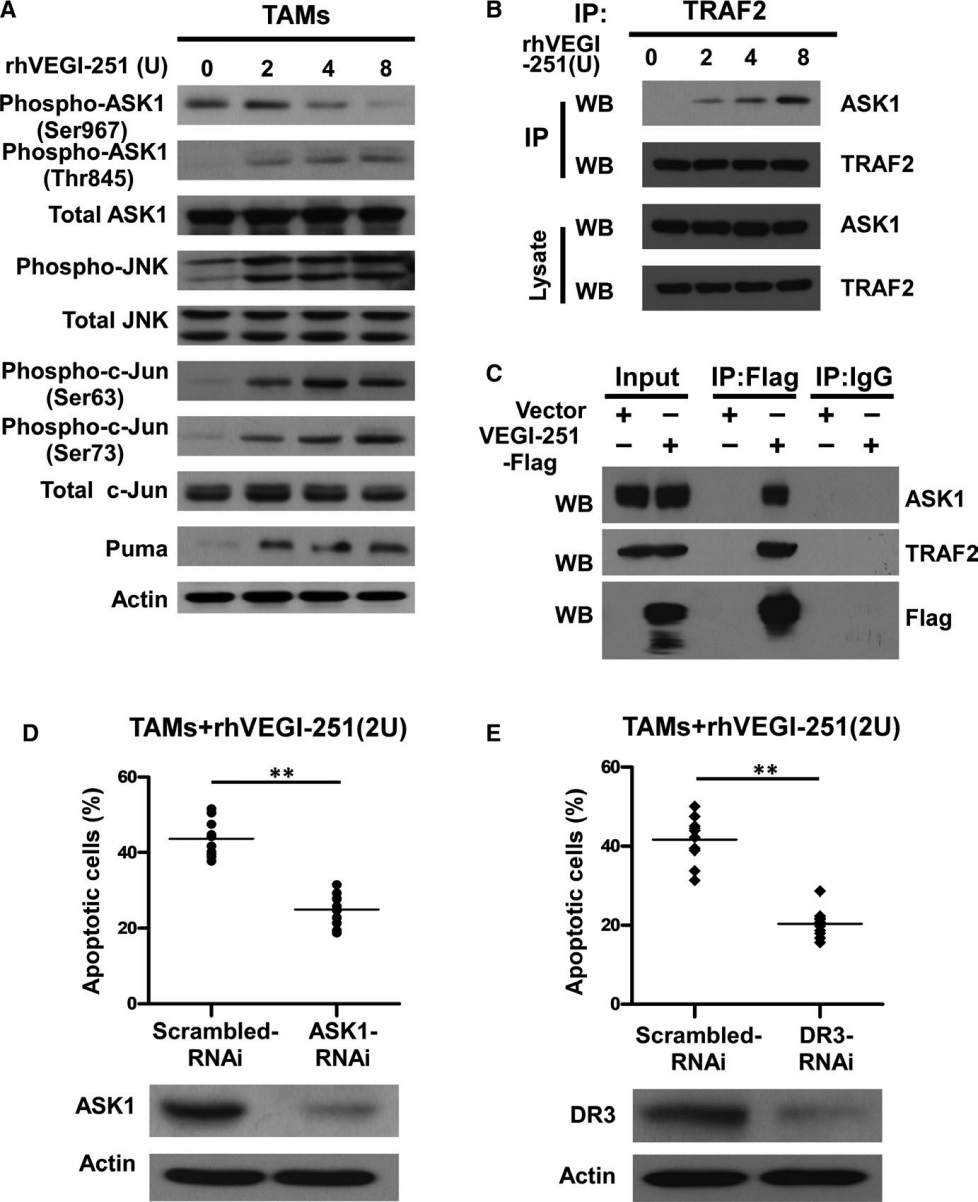
rhVEGI‐251 triggers apoptosis in TAMs in a manner dependent on ASK1 and DR3. A, TAMs were treated with different concentrations of rhVEGI‐251 for 48 h, as indicated. Western blot analysis was performed using indicated antibodies. B, rhVEGI‐251 induced endogenous interaction of ASK1 with TRAF2. TAMs were incubated with varying concentrations of rhVEGI‐251 for 4 h, followed by immunoprecipitation with or without protein G–conjugated anti‐TRAF2 antibody. The immunocomplexes were analysed with anti‐ASK1 or anti‐TRAF2 antibodies by Western blotting. C, VEGI‐251 interacted with ASK1 and the TRAF2 complex. Cell lysates of TAMs with transient overexpression of VEGI‐251 or vector control were immunoprecipitated with the protein G–conjugated IgG antibody (lanes 5 and 6) or anti‐Flag antibody (lanes 3 and 4) followed by immunoblotting. Coimmunoprecipitated ASK1 or TRAF2 protein was detected with the anti‐ASK1 antibody (top) or anti‐TRAF2 antibody (middle). (D,E) TAMs were transfected with a specific ASK1 or DR3 siRNA or negative control siRNA at a final concentration of 50 nmol/L. The protein levels of ASK1 and DR3 were measured by Western blotting. After 24 h of transfection, the cells were treated with rhVEGI‐251, and a TUNEL assay was used to estimate the percentage of apoptotic cells. The apoptotic index was determined by counting the number and calculating the percentages of TUNEL‐positive cells in 10 fields. This experiment was performed for three times with similar results. Data accorded with normal distribution for the quantitative assessment of apoptosis are expressed as the means ± SD Comparisons between two group were made by a two‐tailed Student's *t* test. **P < 0.01 indicates a significant difference compared with control cells

It is known that VEGI can directly activate JNK signalling in the proliferating endothelial cells, which is a cellular event required for VEGI‐192‐mediated apoptosis.^9^, ^12^ To further elucidate the signalling cascade that mediates the pro‐apoptotic effect of rhVEGI‐251 on TAMs, changes in the status of ASK1or JNK phosphorylation and the protein level of its downstream factors, such as c‐Jun and Puma, were determined by Western blotting. As shown in Figure 5A, treatment with the rhVEGI‐251 protein led to a markedly increase in the phosphorylation levels of JNK at Thr183 or Tyr185 in a dose‐dependent manner, but did not affect the expression of total JNK in TAMs. This effect was accompanied by dramatic elevated levels of c‐Jun phosphorylated at Ser63 and Ser73 in a dose‐dependent manner upon treatment with rhVEGI‐251. In parallel with the increase in c‐Jun phosphorylation, the expression of PUMA, a pro‐apoptotic factor, was up‐regulated dose dependently in response to rhVEGI‐251 treatment.

JNK was previously demonstrated to be activated by ASK1, a MAPKKK family member,^26^ which is itself activated through distinct mechanisms in response to various cytotoxic stresses,^26^ such as endoplasmic reticulum (ER) stress, oxidative stress and immune system mediators, including tumour necrosis factor and interleukin‐1.^26^, ^27^, ^28^ To understand the underlying signalling cascade that initiates the pro‐apoptotic effect of rhVEGI‐251, we examined the phosphorylation status of ASK1 at Ser967 and Thr845, as well as the level of total ASK1. As shown in Figure 5A, treatment of TAMs with increasing concentrations of rhVEGI‐251 led to a dose‐dependent decrease in the level of ASK1 phosphorylation at Ser967 and increased the phosphorylation of ASK1 at Thr845 but did not affect the level of total ASK1, indicating the activation of ASK1.

VEGI‐251 has been demonstrated to induce the formation of a DR3 signalling complex containing TRAF2 and to activate the JNK signalling pathway.^16^, ^17^ To understand the molecular mechanisms responsible for the activation of the ASK1/JNK signalling pathway triggered by the VEGI‐251‐DR3 interaction, we examined the association of endogenous TRAF2 and ASK1 in TAMs using a coimmunoprecipitation (co‐IP) assay. Following treatment with rhVEGI‐251 for 4 hours, cell lysates were immunoprecipitated with anti‐TRAF2 monoclonal antibody, and the immunoprecipitates were analysed by incubated with an anti‐ASK1 antibody (Figure 5B). As shown in Figure 5B, ASK1 was associated with TRAF2 in a VEGI‐251‐dependent manner. We also used co‐IP assay to determine the interaction between TRAF2 and ASK1 in TAMs transiently overexpressing VEGI‐251 and confirmed the interaction between TRAF2 and ASK1 was induced by VEGI‐251 (Figure 5C). Taken together, the recruitment of ASK1 to TRAF2 may be responsible for the activation of ASK1/JNK signalling triggered by the VEGI‐251‐DR3 interaction.

### 3.5 VEGI‐251‐induced apoptosis in TAMs requires the VEGI‐251‐DR3 interaction and ASK1 signalling activation

**FIGURE 6:**
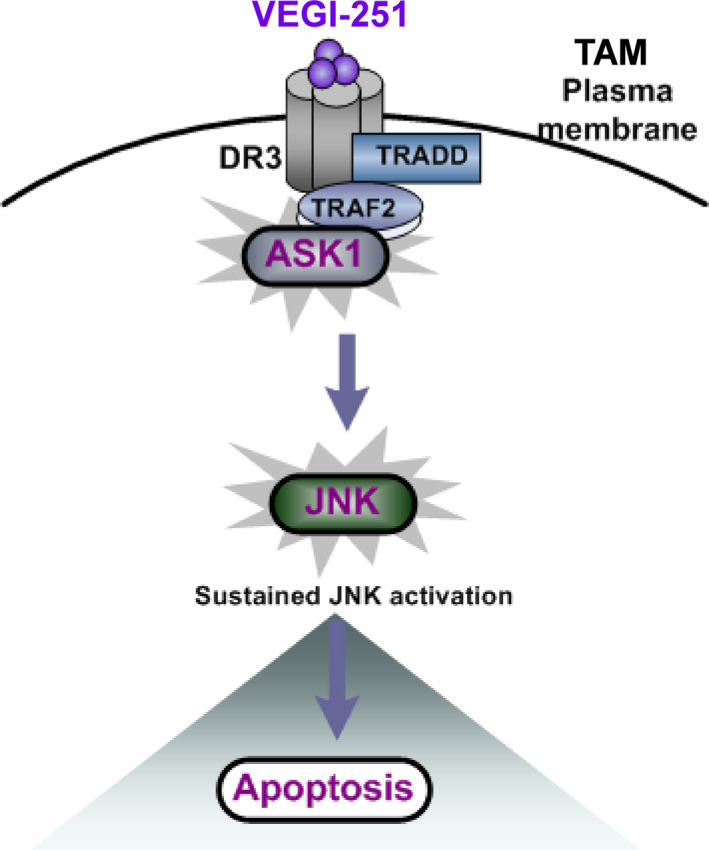
Schematic model of VEGI‐251‐induced TAM apoptosis. VEGI‐251 recognizes DR3 on the membrane of TAMs and recruits TRAF2 and ASK1 to assemble a complex that activates the ASK1/JNK signalling pathway to induce apoptosis through the extrinsic pathway

To understand the main role of ASK1 in the process of TAM apoptosis, ASK1‐specific siRNAs were transfected into purified TAMs to silence ASK1 expression. As shown in Figure 5D, ASK1 protein expression was markedly decreased upon ASK1 siRNA transfection. Notably, depletion of ASK1 resulted in a 24.93 ± 2.04% in VEGI‐251‐induced apoptosis (*P* < 0.05), as compared with that in the vector control‐treated cells (43.69 ± 4.91%) (Figure 5D). Having shown that rhVEGI‐251 could induce TAM apoptosis and enhance ASK/JNK signalling, we further investigated whether DR3, as its target receptor, was involved in this action. To this end, DR3 siRNA transfection successfully produced marked knockdown of DR3 protein expression in TAMs compared to that in macrophages transfected with control retrovirus expressing scrambled siRNA (Figure 5E). As shown in Figure 5E, the percentage of TUNEL‐positive cells was 20.35 ± 3.65% in cells transfected with DR3 siRNA and 41.69 ± 5.90% in control cells under the same conditions (*P* < 0.05). Collectively, these data suggested that VEGI‐251 activates the ASK/JNK pathway in a potentially DR3‐dependent manner and that DR3 can regulate the susceptibility of TAMs to apoptosis induced by VEGI‐251 (Figure 6).

## 4 DISCUSSION

It has been well established that VEGI possesses anticancer abilities through suppressing proliferation of endothelial cells,^10^, ^11^, ^12^, ^13^, ^14^ differentiation of endothelial progenitor cells (EPCs),^29^ angiogenesis and epithelial‐mesenchymal transition. The findings presented in the current report broaden and deepen our knowledge of the diverse strategies developed by VEGI‐251 to suppress tumour growth, including a novel mechanism that targets TAMs. Our results revealed that VEGI‐251 efficiently triggers apoptosis of TAMs in vivo as well as in vitro through the activation of extrinsic apoptotic pathways. In this process, caspase‐8 and caspase‐3 were activated and lead to cleavage of PARP protein and cellular apoptosis. Furthermore, our results showed that VEGI‐251 complexes with DR3, TRAF2 and ASK1 and thus promotes antitumour immune response by triggering TAM apoptosis. Together with the antiangiogenic function of VEGI‐251, these above‐mentioned properties strongly indicate that VEGI‐251 might be a novel therapeutic agent targeting TAMs.

Previous attempts have been made to eliminate TAMs already present in the tumour tissue, suppress the population of pro‐tumoural macrophages in tumour microenvironment through blocking monocyte recruitment, or neutralizing the pro‐tumoural products of TAMs.^2^, ^3^ Our present work revealed a new function of the VEGI‐251 protein, that is to promote the antitumour immune response by inducing apoptotic death of TAMs. In addition, accumulating evidence indicates that augment macrophage polarization towards the M2 phenotype is associated with the progression of many other pathological conditions, including allergic inflammation, asthma, atherosclerosis, Crohn's disease and fibrosis.^30^ Therefore, it will be of great interest to further elucidate whether VEGI‐251 could be a novel therapeutic agent against these M2 macrophage‐associated diseases via a similar mechanism underlying the enhancement of specific pro‐apoptotic effects on M2 macrophages. On the other hand, VEGI‐251 can also enhance the antitumour immune response of lymphocytes. VEGI‐251, as a T‐cell costimulatory factor, can promote the reactivity of T cells for IL‐2 secretion, enhance T‐cell proliferation and induce the production of inflammatory factors, including IFN‐γ and GM‐CSF.^16^, ^31^ Soluble VEGI‐251 can facilitate the proliferation of antigen‐specific CD8^+^ T cells and their differentiation into cytotoxic T cells and can enhance their secondary diffusion.^32^ Furthermore, VEGI‐251 can stimulate the maturation of dendritic cells and induce a Th17 cell–mediated immune response.^33^ Thus, to determine the effect of VEGI‐251 on the enhancement of immune checkpoint therapy and the underlying mechanism in vivo, studies using anti‐PD1 or anti‐PDL1 antibodies combined with systemic administration of VEGI‐251 are ongoing in our laboratory. Taken together, our current study thus raises the question whether the application of rhVEGI‐251‐based strategies could be therapeutically effective in inducing the antitumour immune response in the clinic.

Of note, VEGI‐251 is the only known ligand for DR3.^16^ The expression of DR3 in the cell membrane appears to a range of cells such as T cells, NK cells, lymphocytes and macrophages.^16^ DR3 contains a death domain (DD) in its cytoplasmic region, which can induce cell apoptosis. Our data suggest that VEGI‐251 can markedly trigger TAM apoptosis in vitro and in vivo. In other studies, VEGI‐251 was shown to act as a co‐stimulator of T cells and as a cytokine to induce dendritic cell maturation rather than an inducer of apoptosis.^31^, ^33^ This discrepancy may be due to the difference in the cell types tested; indeed, the model described here is specifically relevant to processes involved in M2 macrophage apoptosis in tumour tissue. Previous reports indicated that the VEGI‐251‐DR3 signalling complex, which consists of TRADD and TRAF2, can transduce signals to activate JNK in cells.^34^ However, the exact interaction between VEGI‐251‐DR3 and ASK1 remains unknown, and a direct crosstalk molecular target of VEGI‐251 that transduces signals to JNK during apoptosis has not been identified. Most importantly, our results demonstrate that activation of the ASK1‐SAPK/JNK pathway in a manner likely dependent on DR3 contributes to VEGI‐251‐triggered TAM apoptosis. To our knowledge, this present study represents the first demonstration that ASK1 may be a key component in the VEGI‐251‐induced JNK activation pathway via recruitment of the DR3‐TRAF2 complex to ASK1 during TAM apoptosis.

## 5 CONCLUSIONS

Taken together, our data suggest that VEGI‐251 can trigger TAM apoptosis and subsequently block the growth of tumours, in addition to its strong suppressive effects on angiogenesis and tumour growth. The VEGI‐251‐DR3 pathway may be an important contributing factor targeting TAMs by aggravating the apoptosis of TAMs through recruiting ASK1 to TRAF2 and activating the ASK‐SAPK/JNK pathway in a potentially DR3‐dependent manner. Thus, TAMs might be a novel target of VEGI‐251 for anticancer therapy, and such treatment method may be effective for several other M2 macrophage‐associated diseases.

## CONFLICT OF INTEREST

The authors declare no conflicts of interest.

## AUTHOR CONTRIBUTIONS

XD, XY, XH, YW, JW, GW and XZ designed experiments; XD, YW, XY, XH, DC, CT, JL, DZ and YH performed the experiments, and analysed the data and contributed reagents/materials/analysis tools; XD, XY, YW, XH, JW, GW and XZ wrote the manuscript. All authors approved the final manuscript.

## Supporting information

Supplementary Material

## Data Availability

The data that support the findings of this study are available from the corresponding author upon reasonable request.
