# Corrigendum: Tumour‐associated macrophages as a novel target of VEGI‐251 in cancer therapy

**DOI:** 10.1111/jcmm.17271

**Published:** 2022-04-05

In Xinhuai Dong et al,^1^ the image data of flow cytometric analysis used as a blank control for 10 mg/kg rhVEGI‐251‐treated group, in the first panel of Figure 3A is incorrect. The correct figure is shown below. The authors confirm that all results and conclusions of this article remain unchanged.

**FIGURE 3:**
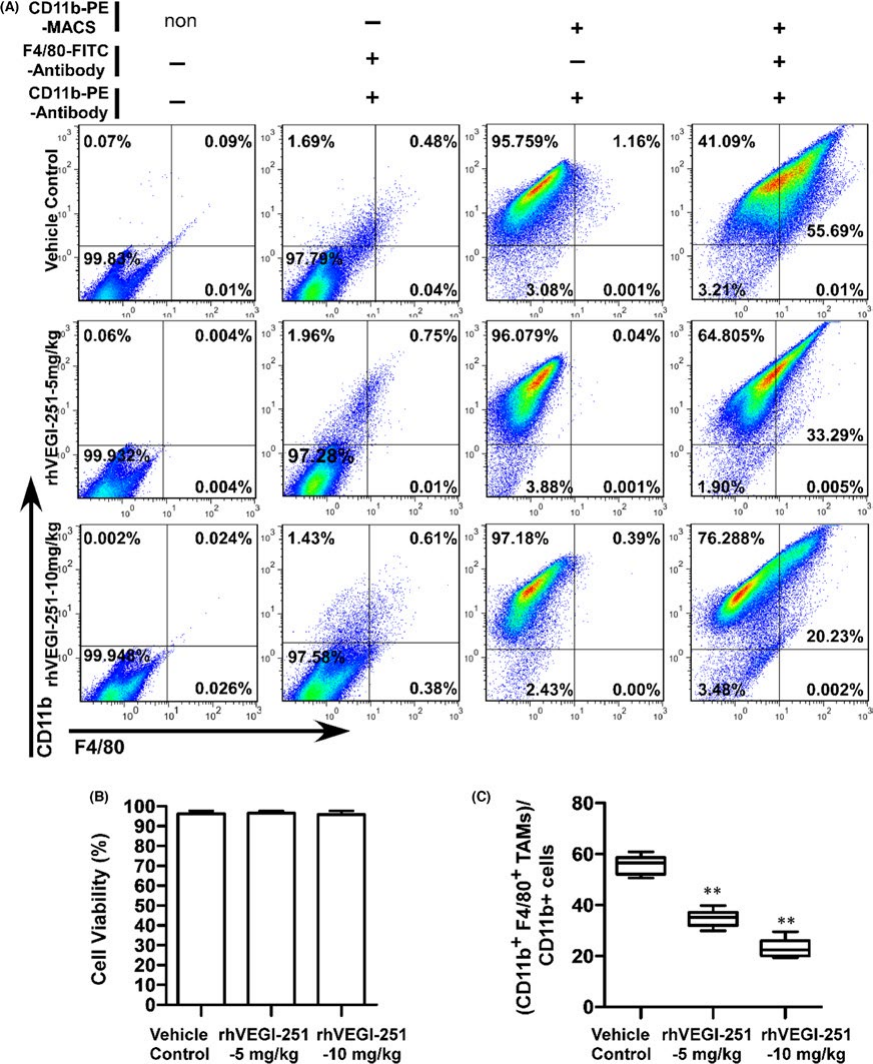
rhVEGI‐251 mediates the elimination of TAMs in tumour tissue. (A) Representative results of flow cytometric analysis of CD11b^+^ F4/80^+^ TAMs from CD11b^+^ tumour‐infiltrating mononuclear cells. The data in the first panel were used as a blank control group, and a symbol ‘non’ on the right of CD11b‐PE‐MACS means there were no CD11b MicroBeads added in this group. The data in the second panel represent the negative control group, and a symbol ‘−‘ on the right of CD11b‐PE‐MACS means these cells were collected as flow‐through (wash fractions) after incubated with CD11b MicroBeads, which are CD11b microbeads negative selected cells. The purity of CD11b^+^ cells among all selected cells is shown in the third panel. The proportions of CD11b^+^ F4/80^+^ cells are shown in the fourth panel. The images are representative of the results from three independent experiments. (B) The viability of purified TAMs was assessed by a trypan blue exclusion assay. (C) Statistical analysis of the percentage of CD11b^+^ F4/80^+^ TAMs among CD11b^+^ tumour‐infiltrating mononuclear cells. One‐way ANOVA followed by Dunnett's multiple comparison test was performed, and significant differences are shown with asterisks (** indicates *p* < 0.01)
