# Supplementary material for: Tumour‐associated macrophages as a novel target of VEGI‐251 in cancer therapy
Source: J Cell Mol Med. 2020 May 26;24(14):7884–95. doi: 10.1111/jcmm.15421 (PMC7348178; doi:10.1111/jcmm.15421)
Supplement: Supplementary file 1 — Supplementary Material [file JCMM-24-7884-s001.docx]

**Supplemental** **Table S1 Sequences of primers used in this work**

|  | Primers | Sequence (5’-3’) |
| --- | --- | --- |
| pEF-BOS-VEGI-251-His | Forward | CGGGGTACCGCCATGGCCGAGGATCTGGGACTGAG |
|  | Reverse | CGCGGATCCTCAGTGGTGGTGGTGGTGGTGTAGTAAGAAGGCTCCAAAG |
| pEF-BOS-VEGI-251-Flag | Forward | CGGGGTACCGCCATGGCCGAGGATCTGGGACTGAG |
|  | Reverse | CGCGGATCCTCACTTATCGTCGTCATCCTTGTAATCTAGTAAGAAGGCTCCAAAG |
| siRNA-ASK1 |  | AAAUGCGUAAUGAAACUUCACGUGG |
| siRNA-DR3 |  | UUCUCCGAACGUGUCACGUTT |
| siRNA-DR3 |  | ACGUGACACGUUCGGAGAATT |

**Supplemental Figure S1**


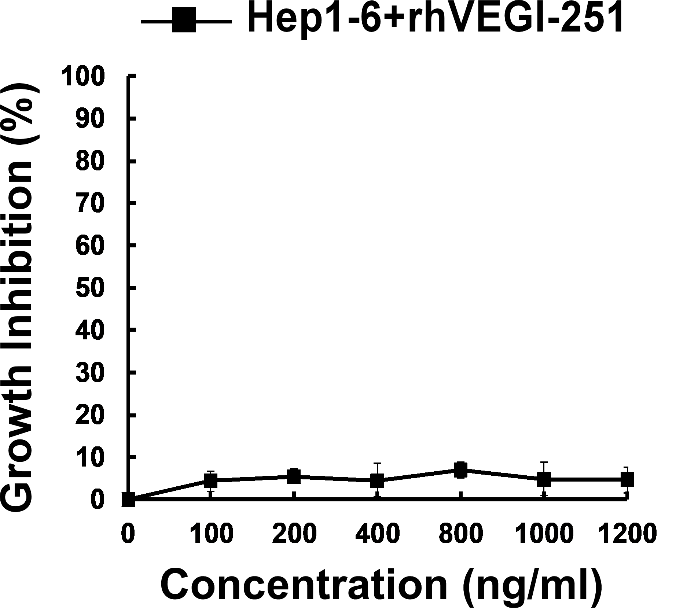


**Figure S1. Effects of rhVEGI-251 on cell viability.** Dose-response curve of the effect of rhVEGI-251 on the proliferation of Hep1-6 after treatment for 48 h. Cell viability was determined by an MTT assay. Results accorded with normal distribution are expressed as mean ± SD of triplicate experiments. One-way ANOVA followed by Dunnett’s multiple comparison test was performed, and significant differences are shown with asterisks (* indicates *p* < 0.05, and ** indicates *p* < 0.01).

**Supplemental Figure S2**


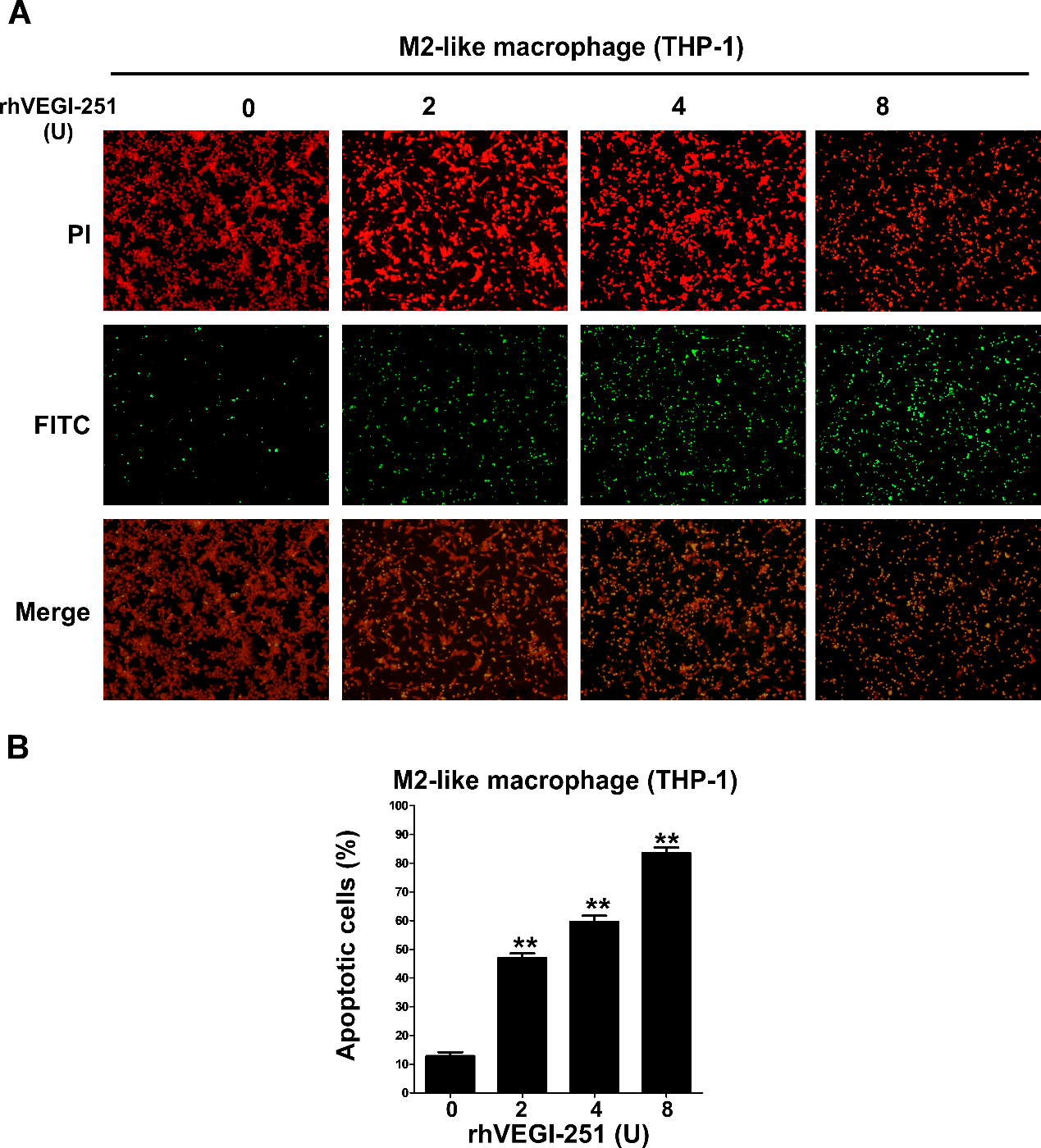


**Figure S2. rhVEGI-251 triggers marked apoptosis of** **M2 macrophages.** (A) Human M2-like macrophages that derived from THP-1 cell lines were treated with different concentrations of rhVEGI-251 as noted for 24 h and were then labeled with fluorescein-12-dUTP (green) and counterstained with PI (red). (B) Apoptotic index, as determined by counting the number and calculating the percentages of TUNEL-positive cells in 10 fields. One-way ANOVA followed by Dunnett's t test was performed. ***p* < 0.01 indicates a significant difference compared with control cells.
